# Is a Parry Fracture—An Isolated Fracture of the Ulnar Shaft—Associated with the Probability of Abuse in Children between 2 and 16 Years Old?

**DOI:** 10.3390/children8080650

**Published:** 2021-07-28

**Authors:** Kyra Hermans, Duncan Fransz, Lisette Walbeehm-Hol, Paul Hustinx, Heleen Staal

**Affiliations:** 1 Department of Trauma Surgery, Zuyderland Medical Center, 6419 PC Heerlen, The Netherlands; ky.hermans@zuyderland.nl (K.H.); hustinxpaul@gmail.com (P.H.); 2 Department of Orthopaedic Surgery, Maastricht University Medical Center, 6229 HX Maastricht, The Netherlands; h.staal@mumc.nl; 3 Department of Orthopaedic Surgery, Zuyderland Medical Center, 6419 PC Heerlen, The Netherlands; 4 Department of Pediatrics, Zuyderland Medical Center, 6419 PC Heerlen, The Netherlands; l.hol@zuyderland.nl

**Keywords:** non-accidental trauma, self-defense, violence, adolescent, dexterity, biomechanics

## Abstract

A parry fracture is an isolated fracture of the ulnar shaft. It occurs when the ulna receives the full force of an impact when the forearm is raised to protect the face. The aim of this study is to assess a possible association between a parry fracture and the probability of abuse in children. In this retrospective, observational, multicenter study, we identified patients between 2 and 16 years old who had been treated for an isolated ulnar shaft fracture. Patient characteristics were registered, anonymized radiographs were rated, and charts were screened for referral to a child protective team. A total of 36 patients were analyzed. As no referrals were registered during follow-up, the primary outcome was changed to a perpendicular force as trauma mechanism. Univariable regression analysis and independent *t*-test both showed no significant association between patient factors or radiographic classification, and the reported trauma mechanism. We were unable to determine an association between a parry fracture and the probability of abuse. Since trauma mechanism does have a biomechanical effect on the fracture type, we would advise that a very clear reconstruction (and documentation) of the trauma mechanism should be established when a parry fracture is identified on radiographs.

## 1. Introduction

**Figure 1:**
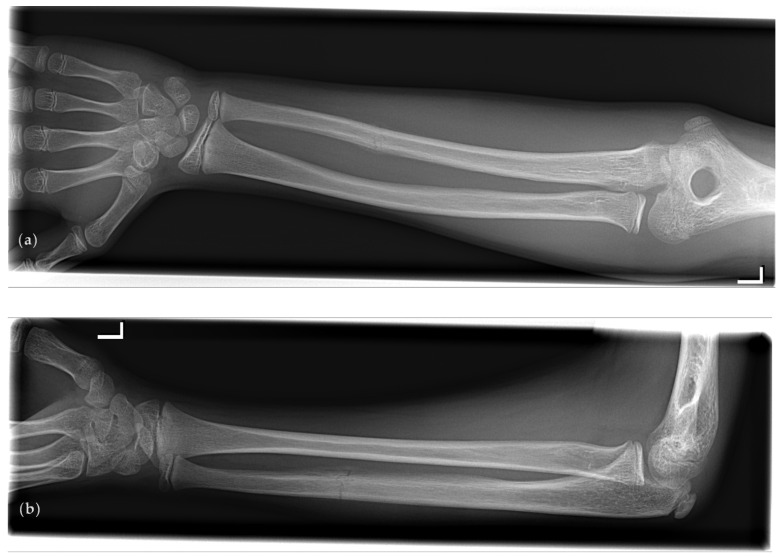
A typical example of an isolated fracture of the left ulna that meets all four parry fracture criteria according to Judd [1]: absence of radial involvement, transverse fracture line, distal from midshaft, and minimal displacement. Radiographs are shown in (**a**) anteroposterior projection; (**b**) lateral projection.

A very specific type of forearm fracture, a so-called ‘parry’ (or ‘nightstick’) fracture, has been associated with interpersonal and extramural violence in the field of paleopathology [1]. A parry fracture is an isolated fracture of the ulnar shaft (see Figure 1) and can occur when the ulna (which is more exposed than the radius when arms are raised as a means of defense) receives the full force of a blunt force attack [2]. Due to the mechanism of trauma that is required to cause such a fracture, it might therefore be associated with (child) abuse.

The proportion of fractures attributed to abuse is highest in infants younger than 1 year (24.9%). This proportion decreases to 7.2% in children 12 to 23 months and 2.9% in children 24 to 35 months of age [3]. Recognizing abusive injuries is critical to preventing further injury and even death of the child [4]. Failure to identify child abuse at the time of initial presentation leaves the victim with a 30–50% chance of recurrent abuse [5].

Unfortunately, distinguishing between non-inflicted fractures and those caused by abuse can be challenging, particularly in children and even more so when they are older than two years. The dependency relationship with the perpetrator ensures that it is difficult to determine whether there is abuse or not. When children are able to relate the situation, there is a fair chance that they will keep silent out of loyalty to the parents or out of fear of the perpetrator [6].

No specific type of fracture, on its own, can distinguish an abusive from a non-abusive cause [6,7]. However, various red flags regarding fracture characteristics have been proposed. These include presentation to the emergency department with multiple fractures, fractures in various states of healing, metaphyseal corner fractures, long bones fractures in children who are not of walking age, spine fractures, scapular fractures, rib fractures, epiphyseal separations, swelling not proportional to injury type (i.e., less swelling than would be expected for an acute fracture), and fractures reported as “falls from a bed or couch” in those less than one year of age [7,8].

When specifically looking into forearm fractures, fractures of the radius and/or ulna are uncommon in infants because they cannot meet the conditions necessary to cause this fracture [3]. In an older child, forearm fractures most often occur from falling onto an arm that is outstretched to break the fall [3]. In a retrospective study on 47 children (2–17 years old) with a forearm fracture that had been screened by a child protective team, no particular type of forearm fracture was inherently indicative of child abuse [9].

However, thus far, no study has investigated a possible association between a parry fracture and abuse in children. Therefore, we set out to analyze a possible relation between isolated ulnar shaft fractures and the probability of abuse in patients between 2 and 16 years old.

## 2. Materials and Methods

### 2.1. Data Collection

For this retrospective study, we identified patients between 2 and 16 years old who were treated for a forearm fracture at the emergency department (ED) or outpatient clinic (OC) at Zuyderland Medical Center or at Maastricht University Medical Center between 1 January 2008 and 1 January 2018.

#### 2.1.1. Radiographs

We retrieved radiology reports for the identified patients, and for those reports that described an isolated fracture of the ulna, the corresponding radiographs were collected. Patients with fractures of the Monteggia type, or with ulnar shaft fractures that were accompanied by any type of radial fracture, were excluded.

#### 2.1.2. Patients

For the remaining patients, we retrieved patient characteristics from their charts. Age at presentation, sex, fractured side, previous fractures, presentation location (ED or OC), trauma mechanism (as described by patient or parent), and delay (days since trauma) were registered. Patients were excluded if osteogenesis imperfecta (brittle bone disease) or any other bone metabolism disorder had been previously diagnosed.

As primary outcome, we recorded if patients had been referred to a child protective team for further evaluation up to 1 January 2021.

### 2.2. Data Analysis

Three assessors (trauma surgeon, pediatric orthopedic surgeon, and pediatrician) reviewed and scored all data individually.

#### 2.2.1. Radiographic Classification

The classification of anonymized radiographs was done according to the AO Pediatric classification [10] and to the paleopathology parry (P3) fracture criteria [1]. If a fracture met all four of the below criteria, it was regarded as a P3 fracture:

1. the absence of radial involvement;
2. a transverse fracture line (≤45°);
3. a location below the midshaft (<0.5 adjusted distance to the lesion’s center); and
4. either minor unalignment (≤10°) in any plane or horizontal apposition from the diaphysis (<50%).

Classification was done twice, the second time after a repeated randomization.

#### 2.2.2. Chart Assessment

None of the included patients had been referred to a child protective team for further evaluation. Therefore, we had to formulate an alternative primary outcome. As any retrospective interpretation of the available information on an ED or OC chart would introduce several forms of bias, we decided to select the reported trauma mechanism as the primary outcome. If the chart stated that the child fell (from an object or during an activity), we regarded the trauma mechanism as a parallel force. If the chart stated that someone exerted a force upon the arm, we regarded the trauma mechanism as a perpendicular, direct force. 

### 2.3. Statistical Analysis

All statistical analyses were performed using IBM SPSS Statistics for Mac (IBM Corp., version 21.0, Armonk, NY, USA).

For each radiograph, a resultant classification was compiled from the 3 (assessors) × 2 (repetitions) assessments. If the resultant remained inconclusive (i.e., majority rule), the assessment of a fourth assessor served as the decider. To assess the agreement within and between the assessors regarding the different radiographic classifications, we calculated Cohen’s kappa [11]. This method corrects for agreement based on chance and is well suited for categorical variables. A value above 0.6 is regarded as substantial agreement.

We used univariable binary logistic regression analysis to quantify the association between potential determinants (patient characteristics and fracture classifications) and the dependent variable (an evident perpendicular force as trauma mechanism). The patient characteristics ‘age’ and ‘delay’ were entered as continuous variables, all others were entered as dichotomous. Though strictly not a continuous variable, we did consider the AO Pediatric classification as continuous (in contrast to categorical), since a ‘higher’ classification essentially implies a more serious injury. Whether or not a parry fracture was apparent was entered as a dichotomous variable.

We also compared mean values between the two groups (i.e., fall versus direct trauma) with an independent samples *t*-test. For both statistical tests, we considered a *p*-value < 0.05 as an indication of statistical significance.

## 3. Results

### 3.1. Data Collection

Between 1 January 2008 and 1 January 2018, a total of 42 patients were treated at the ED or OC for an isolated ulna shaft fracture. Six patients had to be excluded from further analysis: five patients did not have any information at all in their charts, however the radiographs and radiology reports were available; one patient had missing radiographs. The patient characteristics for the 36 included patients are shown in Table 1, an overview of the various reported trauma mechanisms is shown in Table 2.

### 3.2. Data Analysis

In Table 3, the results for the fracture classifications as evaluated by the assessors are shown. For both classifications, assessment by a fourth assessor was necessary in three cases to break the tie.

As none of the patients had been referred to a child protective team for further evaluation by the 1 January 2021, we decided to select the reported trauma mechanism as the primary outcome. In Table 2, the distinction between a parallel (fall, *n* = 30) and an evident perpendicular force (direct, *n* = 6) is apparent.

### 3.3. Statistical Analysis

The agreement within and between assessors is available as Supplemental Data. The intra-observer agreement was higher than the inter-observer agreement. In general, the agreement between assessors was fair to moderate [12], with the P3 criteria scoring higher than the AO Pediatric classification.

Regression analysis showed no significant association between patient characteristics and fracture classifications, and an evident perpendicular force as trauma mechanism (Table 4). Similarly, the *t*-test did not show a significant difference between group means (Table 5).

## 4. Discussion

In this study, we were unable to determine an association between a parry fracture and the probability of abuse. We selected our patient group based on fracture type, and as it turned out, this group did not include children who had been referred to a child protective team for further evaluation during the follow-up period.

This finding is in accordance with a previous study that showed that no particular type of forearm fracture was specific for abuse in children younger than 18 months [9]. However, while not addressing the parry fracture specifically, transverse fractures were seen in 45% (5/11) of the abusive fractures, compared to 28% (9/32) of non-inflicted fractures [9].

As our group sizes were small, no statistical difference was expected to be found. However, it seems that age, side, and the P3 criteria are somewhat related to the trauma mechanism, albeit non-significantly (Table 4 and Table 5).

In general, specific fracture types are caused by a particular application of force. Transverse fractures are caused by a bending load perpendicular to the bone, spiral fractures by torsion along its long axis, and oblique fractures by a combination of both [13]. The reported direct mechanisms (Table 2) are comparable to a direct blow when the forearm is raised to protect the face, as is the case with a parry fracture [1]. Therefore, we would advise that a very clear reconstruction of the trauma mechanism should be established, especially if a parry fracture is identified on a radiograph. Even more importantly, these details should be written down in the charts. We found that the limited information for both medical history and physical examination highlighted another known problem: the ED documentation of pediatric injury is quite insufficient, making child abuse very difficult to suspect [14].

The possible effect of age is most likely due to the different activities older kids undertake, predisposing them to this trauma mechanism.

The current study showed 58% of ulnar fractures on the left side, which was to be expected in the case of a fall. Children tend to favor their left hand to protect themselves when they fall; sometimes the dominant hand is engaged in some activity. The left arm also seems to fracture more easily because of greater fragility, immaturity, and suboptimal neuromuscular coordination, rendering the left arm less suited to managing the situation [15]. However, in the case of abuse, the left side also fractures more often. In a study on a total 124 unclaimed cadavers prone to abuse, the left ulna was the most affected long bone of the upper limb. This may be explained by a right-sided attack [2]. 

This study had several limitations. In contrast to a previous study [9], the basis of our study was a specific type of forearm fracture. As it turned out, our sample did not include any cases where actual abuse was identified; none of the patients had been referred to a child protective team for further evaluations. Therefore, we were unable to answer our original research question, though we had a minimum follow-up of three years. However, the follow-up was limited to the hospital of initial presentation and subsequent presentation to another hospital therefore was not considered.

Even though the isolated ulnar shaft fracture is an uncommon fracture type (4% of the forearm fractures in children) [9], the current sample of 36 patients is perhaps still too small to identify associations between type of fracture and possible child abuse. A larger patient population would be desirable.

Since absence of proof is not proof of absence, future research should focus on identifying as many red flags regarding child abuse as possible. Perhaps the advent of machine learning and big data can further assist in this multifactorial domain of relatively low incidence but grave consequences. Failing to recognize abuse at the initial presentation leaves the victim with a 30–50% chance of recurrent abuse, as well as an increased risk of morbidity and mortality [5].

## 5. Conclusions

We were unable to determine an association between a parry fracture and the probability of abuse. If, however, the described trauma mechanism consisted of a perpendicular bending load, the resulting fracture often met the criteria for a parry fracture. Therefore, we would advise that a very clear reconstruction (and documentation) of the trauma mechanism should be established when a parry fracture is identified on radiographs.

## Figures and Tables

**Table 1:** Characteristics of the included patients (*n* = 36).

Determinant	*n* (%)	mean ± *SD* (Range)
Age (years)		8.9 ± 3.8 (2.3–15.4)
Sex (male)	23 (64%)	
Side (right)	15 (42%)	
Previous fracture (yes)	3 (8%)	
Delay (yes)	8 (22%)	9.3 ± 3.9 (1–14) *

* The mean ± *SD* when there was a delay in presentation of the fracture, in days.

**Table 2:** Overview of reported trauma mechanisms (*n* = 36).

**Fall from**	***n***	**Fall during**	***n***
bicycle	1	cartwheeling	1
bouncy castle	2	dancing	1
chair	1	field hockey	1
climbing frame	3	gymnastics	2
couch	1	handball	1
gymnastic vault	2	playing with old rubber tire	1
hoverboard	1	playing in the mud	1
pony	1	soccer	1
sandbox	1		
sidewalk	1	**Direct Trauma**	***n***
slide	2	kicked by other kid	2
small pole	1	kicked by pony	1
standing	1	other kid fell on arm	1
swing	1	other kid stepped on arm	2

In two cases, there was notion of a fall, but without a cause.

**Table 3:** Fracture classifications and the contributing assessments.

Classification		Contributing Assessments (%)	
AO Pediatric	*n*	yes	no
Bowing (1.1)			
Greenstick (2.1)	9	69	31
Complete transverse 1 (4.1)	10	75	25
Complete transverse 2 (4.2)			
Complete oblique or spiral 1 (5.1)	15	68	32
Complete oblique or spiral 2 (5.2)	2	75	25
Paleopathology			
Parry fracture	14	77	23
No parry fracture	22	89	11

Percentages were calculated based on 3 (assessors) × 2 (repetitions) × *n* assessments. For instance, of all the fractures that were classified as a ‘Complete transverse 1′ according to the AO Pediatric (i.e., *n* = 10), 25% of the assessments did not classify the fracture as such. Assessment by a fourth assessor was necessary in three cases for each the AO Pediatric classification (this resulted in 2 × 5.1 and 1 × 4.1) and the paleopathology criteria (2 × yes, 1 × no).

**Table 4:** Univariable binary logistic regression analysis.

	Exp(B) [95% C.I.]	*p*-Value
Age	1.289 [0.980–1.695]	0.069
Sex	1.158 [0.182–7.384]	0.877
Side	0.229 [0.240–2.198]	0.201
Previous fracture	2.800 [0.212–37.03]	0.434
Delay	1.029 [0.846–1.251]	0.778
AO Pediatric	1.055 [0.554–2.011]	0.870
Paleopathology	4.667 [0.720–30.23]	0.106

Age (years), delay (days), and AO Pediatric classification were entered as continuous variables. The other variables were entered as dichotomous; sex (0 = female), side (0 = left), previous fracture (0 = none), and paleopathology criteria (0 = no).

**Table 5:** Means, standard deviation, and *p*-value when comparing groups of the patients that suffered from a fall with patients that suffered from a direct impact.

	Fall (*n* = 30)	Direct (*n* = 6)	*p*-Value
Age	8.41 ± 3.78	11.71 ± 3.10	0.053
Sex	0.63 ± 0.49	0.67 ± 0.52	0.881
Side	0.47 ± 0.51	0.17 ± 0.41	0.183
Previous fracture	0.07 ± 0.25	0.17 ± 0.41	0.433
Delay	1.97 ± 4.06	2.50 ± 5.65	0.784
AO Pediatric	3.73 ± 1.34	3.83 ± 1.72	0.874
Paleopathology	0.30 ± 0.47	0.67 ± 0.52	0.093

Age (years), delay (days), and AO Pediatric classification were entered as continuous variables. The other variables were entered as dichotomous; sex (0 = female), side (0 = left), previous fracture (0 = none), and paleopathology criteria (0 = no).

## Data Availability

Not applicable.

## Supplementary Materials

The following are available online at https://www.mdpi.com/article/10.3390/children8080650/s1: Table S1: Assessor agreement for the radiographic classifications.
