# Supplementary material for: Is a Parry Fracture—An Isolated Fracture of the Ulnar Shaft—Associated with the Probability of Abuse in Children between 2 and 16 Years Old?
Source: Children (Basel). 2021 Jul 28;8(8):650. doi: 10.3390/children8080650 (PMC8394848; doi:10.3390/children8080650)
Supplement: Supplementary file 1 [file children-08-00650-s001.zip › children-1267275-supplementary.pdf]

Table S1. Assessor agreement for the radiographic classifications

	AO Pediatric	Paleo-pathology
Within		
X1 vs X2	0.541	0.585
Y1 vs Y2	0.386	0.640
Z1 vs Z2	0.502	0.833
Between		
X1 vs Y1	0.413	0.137
X2 vs Y2	0.275	0.348
X1 vs Z1	0.448	0.373
X2 vs Z2	0.609	0.556
Y1 vs Z1	0.333	0.494
Y2 vs Z2	0.289	0.444

Cohen's kappa corrects for agreement based on chance and is well suited for categorical variables. A value above 0.6 is regarded as substantial agreement.
